# Long-Term Mortality of Patients with Septic Ocular or Central Nervous System Complications from Pyogenic Liver Abscess: A Population-Based Study

**DOI:** 10.1371/journal.pone.0033978

**Published:** 2012-03-27

**Authors:** Yi-Tsung Lin, Chia-Jen Liu, Tzeng-Ji Chen, Chang-Phone Fung

**Affiliations:** 1 Division of Infectious Diseases, Department of Medicine, Taipei Veterans General Hospital, Taipei, Taiwan; 2 Division of Hematology and Oncology, Department of Medicine, Taipei Veterans General Hospital, Taipei, Taiwan; 3 School of Medicine, National Yang-Ming University, Taipei, Taiwan; 4 Department of Family Medicine, Taipei Veterans General Hospital, Taipei, Taiwan; The University of Melbourne, Australia

## Abstract

**Background:**

Taiwan is endemic for pyogenic liver abscess (PLA). Septic ocular or central nervous system (CNS) complications derived from PLA can result in catastrophic disability. We investigated the epidemiology and long-term prognosis of PLA patients with septic ocular or CNS complications over an 8-year period.

**Methodology/Principal Findings:**

We extracted 21,307 patients with newly diagnosed PLA from a nationwide health registry in Taiwan between 2000 and 2007. The frequency of and risk factors for PLA with septic ocular or CNS complications were determined. The 2-year survival of these patients was compared between those with and without septic ocular or CNS complications. Septic ocular or CNS complications accounted for 2.1% of all PLA patients. Age and the Charlson comorbidity index were significantly lower in PLA patients with ocular or CNS complications than those without. Diabetes and age <65 years were independent predictors of septic ocular or CNS complications. The 2-year mortality of patients with septic ocular or CNS complications was similar to those without complications (24.8% vs. 27.5%, p = 0.502). However, among patients <65 years old and a Charlson index ≤1, the 2-year mortality was significantly higher in those with than without complications (18.6% vs. 11.8%, p = 0.001).

**Conclusions/Significance:**

Physicians should recognize that catastrophic disability due to ocular or neurological complications from PLA could lead to a poor long-term prognosis, and should follow-up these patients more closely.

## Introduction

Pyogenic liver abscess (PLA) results from bacterial infection of the liver parenchyma, with subsequent infiltration by inflammatory cells and formation of pus. PLA is associated with significant morbidity, mortality, and health-care costs [1], [2]. The disease is rare in Western countries, for example, the reported annual incidence rate was 2.3 per 100,000 population in Canada [1] 1.0 per 100,000 population in Denmark [3], and 3.6 per 100,000 population in the United States [2]. In Taiwan, however, the annual incidence of PLA has increased steadily from 11.2 per 100,000 population in 1996 to 17.6 per 100,000 population in 2004 [4]. Reported case-fatality rates of PLA from population-based studies in Western countries are substantial, ranging from 5.6 to 19% [1]–[3]. According to a previous population-based study, the overall mortality rate of PLA in Taiwan was 10.9% [4].

*Escherichia coli* is the most common causative pathogen of PLA in most countries [5]. However, in the past three decades, *Klebsiella pneumoniae* has emerged as the single leading cause of PLA in East Asian countries, especially in Taiwan [6]–[13]. A distinct syndrome of community-acquired pyogenic liver abscess, which is complicated by metastatic endophthalmitis or central nervous system (CNS) infections, has been reported since 1986, especially in patients with diabetes [6], [11], [14]–[19]. Despite aggressive therapy, the outcomes of these patients frequently involve catastrophic disability.

A previous study conducted in Taiwan has suggested that metastatic infection predicted mortality [17]. However, another study from a different institute in Taiwan demonstrated a low mortality rate of PLA, even in patients with septic ocular or CNS complications, and suggested that the main concern was no longer mortality, but catastrophic disability due to irreversible ocular or neurological complications [11]. Although Taiwan is endemic for PLA [4], population-based studies evaluating the epidemiology and the long-term outcomes of PLA with septic ocular or CNS complications have never been reported.

In the present study, we used a nationwide population-based database to investigate the frequency of, risk factors for, and long-term mortality of PLA with septic ocular or CNS complications over an 8-year period in Taiwan.

## Materials and Methods

### Database

This study was based on data from the National Health Insurance (NHI) Research Database released by the National Health Research Institute. Taiwan began its National Health Insurance program in 1995 to finance health care for all of its residents. There are currently more than 25 million enrollees in the program, representing approximately 99% of Taiwan's entire population. The database includes comprehensive information on insured subjects, such as demographic data, dates of clinical visits, diagnostic codes, details of prescriptions, and expenditure amounts. International Classification of Diseases, Ninth Revision codes were used to define diseases during the study period. Laboratory data and microbiological data are not included. The highly trustworthy National Health Insurance Research Database has been used in the research of PLA published in peer-reviewed journals [4], [20]. The dataset used in this study consists of de-identified secondary data released to the public for research purposes. This study was approved by the institutional review board of Taipei Veterans General Hospital. Since the identification numbers and personal information of all individuals were not included in the above secondary files to protect the privacy of the individuals, the review board approved that written consents from patients were not required.

### Study Population

In this retrospective cohort study, we identified 24,897 patients who had been hospitalized due to a diagnosis of liver abscess (ICD-9-CM diagnosis code 572.0) between January 1, 2000 to December 31, 2007. Amebic liver abscess (ICD-9-CM diagnosis code 006.3) was not included in this study because of its distinct features and etiologies. Patients with a past history of liver abscess prior to January 1, 2000 (n = 510), under the age of 18 years (n = 222), with underlying hepatobiliary malignancies or hepatobiliary malignancies diagnosed within 60 days (ICD-9-CM 155–156.9) (n = 2868) were excluded [20]. The final sample included 21,297 patients. The underlying diseases and operative procedures, such as liver resection and drainage, were also collected for analysis. Comorbidities were recorded and used to obtain the Charlson comorbidity index [21], [22].

PLA patients with septic ocular complications were defined as PLA patients with purulent endophthalmitis (ICD-9-CM diagnosis code 360.0–360.04) or those receiving a vitrectomy (ICD-9-CM procedural codes 14.7–14.79) during the same admission. PLA patients with CNS complications were defined as PLA patients with meningitis (320–320.9 and 322.9) or a brain abscess (324–324.9 and 326) during the same admission.

### Statistical Analyses

Comparisons between groups were made using the χ2 test for categorical variables. All comparison tests were two-sided. Risk factors with *P* values less than 0.1 were entered into a multivariate analysis. A *P* value<0.05 was considered statistically significant. Survival curves were computed by the Kaplan–Meier method and were compared with a log-rank test. All statistical analyses were performed using SPSS statistical software version 17.0 for Windows (SPSS, Inc., Chicago, Illinois).

## Results

During the study period, a total of 21,297 patients with PLA were enrolled. Patients ranged in age from 18 to 101 years, with a median age of 61 years. A total of 8,959 (42.1%) patients had diabetes mellitus, 1,125 (5.3%) had cirrhosis of the liver, 4,484 (21.1%) had cholelithiasis, 1,315 (6.2%) had chronic obstructive pulmonary disease, 1,348 (6.3%) had chronic kidney disease, and 1,405 (6.6%) had extrahepatic malignancies. Male patients dominated the study population (13,104/21,307; 61.5%). The proportions of patients who received abscess drainage and surgical intervention were 28.5% and 11.6%, respectively. The 60-day mortality rate was 7.6%.

The number of identified PLA cases increased from 2,277 in 2000 to 2,911 in 2007 (Table 1). Among all of the patients with PLA, 453 (2.1%) had septic ocular or CNS complications. Specifically, 294 patients had an endogenous endophthalmitis, 176 patients had meningitis or a brain abscess, and 17 patients had both ocular and CNS complications. The annual frequency of PLA with septic ocular or CNS complications was not significantly different throughout the study period (p = 0.068) (Table 1).

**Table 1:** The frequency of PLA cases with septic ocular or central nervous system complications.

Year	PLA with septic ocular or central nervous system complications	Total PLA cases	%
2000	55	2,277	2.4
2001	46	2,423	1.9
2002	69	2,535	2.7
2003	57	2,604	2.2
2004	67	2,778	2.4
2005	54	2,837	1.9
2006	44	2,932	1.5
2007	61	2,911	2.1
Total	453	21,297	2.1

**NOTE.** PLA, pyogenic liver abscess.

The clinical characteristics of PLA patients with and without septic ocular or CNS complications are presented in Table 2. The 60-day mortality rate was not different between the two groups. PLA patients with septic ocular or CNS complications were more likely to be male (66.0% vs. 61.4%; p = 0.051) and younger (56.9±13.3 vs. 60.2±15.2 years; p<0.001) than patients without septic ocular or CNS complications. The Charlson comorbidity index was lower in PLA patients with ocular or CNS complications that without (1.1±1.4 vs. 1.5±1.9; p<0.001). In regards to underlying diseases, there were no significant differences between the two groups in underlying liver cirrhosis and cerebrovascular disease. The prevalence of diabetes was significantly higher among PLA patients with septic ocular or CNS complications (52.1% vs. 41.8%; p<0.001). Conversely, the prevalence of chronic renal disease (4.0% vs. 6.4%; p = 0.045), chronic obstructive pulmonary disease (2.9% vs. 6.2%; p = 0.003), cholelithiasis (6.6% vs. 21.4%; p<0.001), congestive heart failure (1.3% vs. 4.9%; p<0.001), and extrahepatic malignancies (3.8% vs. 6.7%; p = 0.016) was significantly lower among PLA patients with septic ocular or CNS complications.

**Table 2:** Clinical characteristics of PLA patients with and without septic ocular or central nervous system complications.

Characteristics	PLA patients with septic ocular or central nervous system complications (n = 453)		PLA patients without septic ocular or central nervous system complications (n = 20,844)		p value
	Number	%	Number	%	
Gender					0.051
Male	299	66.0	12,805	61.4	
Female	154	34.0	8,039	38.6	
Age					<0.001
<65	230	70.6	12,015	57.6	
≥65	133	29.4	8,829	42.4	
Underlying diseases					
Diabetes mellitus	236	52.1	8,723	41.8	<0.001
Chronic renal failure	18	4.0	1,330	6.4	0.045
Extrahepatic malignancy	17	3.8	1,388	6.7	0.016
Chronic obstructive pulmonary disease	13	2.9	1,302	6.2	0.003
Liver cirrhosis	20	4.4	1,105	5.3	0.454
Cholelithiasis	30	6.6	4,454	21.4	<0.001
Congestive heart failure	6	1.3	1,014	4.9	<0.001
Cerebrovascular disease	39	8.6	2,129	10.2	0.307
Management					
Abscess drainage	149	32.9	5,923	28.4	0.039
Surgical intervention	41	9.1	2,422	11.6	0.105
60-day mortality	41	9.1	1,570	7.5	0.241

**NOTE.** PLA, pyogenic liver abscess.

A binary logistic regression was used to identify the independent risk factors for PLA with septic ocular or CNS complications. Diabetes (odds ratio [OR] = 1.46, 95% CI =  1.21–1.76, *P*<0.001), and age <65 years (OR = 1.51, 95% CI = 1.23–1.86, p<0.001) were independent predictors of septic ocular or CNS complications among all PLA patients. Extrahepatic cancer (OR =  0.61, 95% CI = 0.37–0.99, p  =  0.047), cholelithiasis (OR =  0.29, 95% CI = 0.20–0.43, p<0.001), and congestive heart failure (OR =  0.31, 95% CI = 0.14–0.71, p = 0.005) were associated with a lower risk of septic ocular or CNS complications in PLA patients.

**Figure 1:**
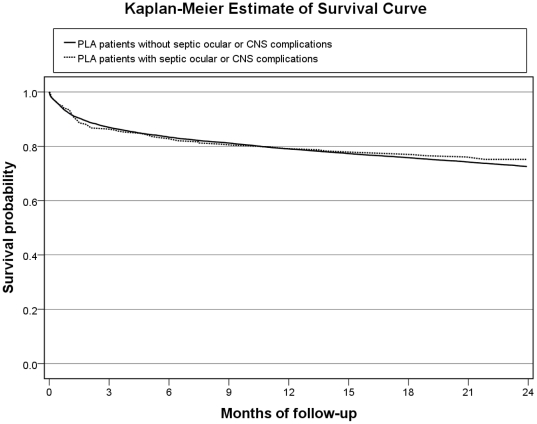
A comparison of 2-year Kaplan–Meier survival curves of PLA patients with and without septic ocular or CNS complications (p = 0.502, by log-rank test).

**Figure 2:**
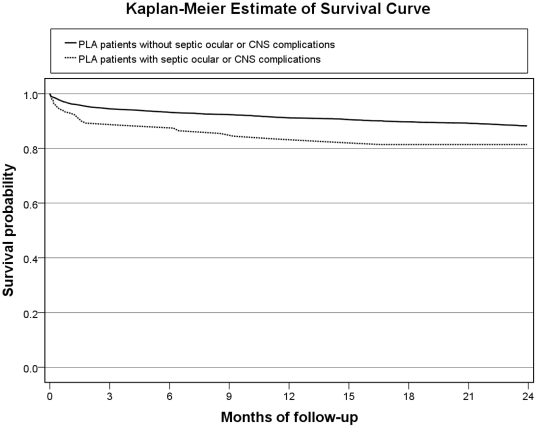
A comparison of 2-year Kaplan–Meier survival curves of PLA patients aged <65 years and a Charlson comorbidity index ≤1 (i.e. between 0 and 1) with and without septic ocular or CNS complications (p = 0.001, by log-rank test).

The Kaplan-Meier survival curves of PLA patients with and without septic ocular or CNS complications are presented in Figure 1. There was no significant difference in the 2-year mortality between PLA patients with and without septic ocular or CNS complications (24.8% vs. 27.5%, p = 0.502, by log-rank test). We further selected PLA patients <65 years old and a Charlson comorbidity ≤1 (i.e. between 0 and 1) for further comparison (249 patients with complications and 9082 without complications), and the 2-year mortality was significantly higher among PLA patients with septic ocular or CNS complications than those without (18.6% vs. 11.8%, p = 0.001, by log-rank test) (Figure 2).

## Discussion

The present study is a first ever nationwide population-based study to report on the epidemiology and long-term outcomes of PLA with septic ocular or CNS complications in Taiwan. The strengths of the present study are that it is based on a large nationwide population-based sample, contains data on a wide variety of demographic characteristics, and has a complete history of the medical service used by the sampled patients. We found that 2.1% of all PLA cases presented with septic ocular or CNS complications. It should be noted that the annual frequency of septic ocular or CNS complications was relatively steady throughout the study period. Although management of PLA has improved in Taiwan due to increased physician awareness [4], it is still likely that complications arise at the time of presentation, and thereby, are not preventable, regardless of the antibiotic treatment used [17]. Therefore, it is essential for physicians to maintain high clinical vigilance for the development of these metastatic infections through early detection and management of high-risk patients in the endemic region.

Investigations comparing the clinical features of PLA patients with and without septic ocular or CNS complications have been rarely reported [17]. The risk factors for septic metastatic infections include the virulence factors of *K. pneumoniae* [11] and host factors, such as diabetes [18]. The current nationwide population-based study focused on host factors and demonstrated that individuals with diabetes and an age <65 years are predisposed to metastatic infection via multivariate analysis. The concept that younger individuals are more susceptible to metastatic infection has never been reported, in part, due to the small sample sizes of patients in the literature [11], [17], [18]. As for other demographic data, extrahepatic cancer, cholelithiasis, and congestive heart failure were all associated with a lower risk of septic ocular or CNS complications. All of these findings suggest that septic ocular or CNS complications are more likely to develop in younger and diabetic, but otherwise healthy, patients.

In one previous study, 70% of patients with septic ocular or CNS complications experienced irreversible catastrophic disability, including loss of vision in the affected eye(s), quadriplegia, paraparesis, or impaired higher cortical function [11]. Poor visual outcomes, despite aggressive management, were also documented [16], [18], [23]. However, the long-term mortality of patients with septic ocular or CNS complications has not yet been investigated. The short-term mortality (i.e. 60-day) was slightly higher in PLA patients with septic ocular or CNS complications, however not statistically significant (9.1% vs. 7.5%; p = 0.241). It may be reasonable to suggest that the infectious process *per se* was the dominant cause of short-term mortality. However, irreversible catastrophic disability from septic ocular or CNS complications may lead to mortality in a few months to years later. Recognizing the potential for adverse long-term outcomes is important, as the findings from previous studies that evaluated only the short-term mortality likely underestimated the true burden of the illnesses associated with PLA.

It is well-known that co-morbid illnesses and age have an important influence on long-term mortality of infectious diseases [24], [25]. The older age and co-morbidities may contribute to the long-term mortality significantly in PLA patients without septic ocular or CNS complications. Although PLA patients with complications were younger and had a lower Charlson cormobidity index than those without complications, the complications may lead to the similar long-term outcome between the 2 groups. The long-term mortality following infection may be attributable to a combination of pre-existing co-morbidities, the nature and severity of the initial infection, and the direct complications arising from this acute disease. Given the lower number of co-morbidities and the slightly higher short-term mortality, the long-term mortality of PLA patients with septic ocular or CNS complications may be attributed to irreversible catastrophic disability. To clarify the poor long-term prognosis of PLA patients with septic ocular or CNS complications, PLA patients <65 years old and a Charlson comorbidity index ≤1 were selected for further analysis. The result demonstrated that these patients have a lower 2-year survival than those without septic ocular or CNS complications. Thus, physicians should be informed about the distinct clinical group of PLA patients that are younger and have less co-morbidities, and simultaneously carry a poor long-term prognosis.

This study suffers from several limitations. First, the study does not have microbiological data, however, it is well established that *K. pneumoniae* is the major etiological factor of PLA in Taiwan, and is exclusively responsible for septic ocular or CNS complications [11]. Second, detailed clinical variables, including clinical manifestations and laboratory findings, were not available. Consequently, we were not able to directly assess the relationship between the severity of disease and long-term mortality. Lastly, we did not include quality of life measurement, which are important markers of long-term outcome following PLA.

In conclusion, this study provides a clear picture of with respect to the disease burden of PLA with septic ocular and CNS complications in Taiwan. Our findings indicate that septic ocular or CNS complications accounts for 2.1% of PLA patients in Taiwan. Septic ocular or CNS complications are more likely to develop in younger and diabetic, but otherwise healthy, patients. Physicians should recognize that catastrophic disability due to ocular or neurological complications from PLA could lead to a poorer long-term prognosis, and thereby, follow up these patients more closely. A comprehensive and collaborative management of PLA via the combined efforts of multiple medical subspecialties is necessary to tackle this problem in the future.
